# Development and Preliminary Application of a Beak Development Index in Pathogenicity Studies of Novel Goose Parvovirus

**DOI:** 10.3390/vetsci12121152

**Published:** 2025-12-02

**Authors:** Yu Shao, Dong Huang, Zhanjie Han, Yanfang Cong, Jinliang Wang, Xiaobin Wang, Ruimei Yang, Shijun Bao, Hongliang Zhang

**Affiliations:** 1 College of Veterinary Medicine, Gansu Agricultural University, Lanzhou 730070, China; 107332214001@st.gsau.edu.cn (Y.S.); 107332214002@st.gsau.edu.cn (D.H.); 2 Shandong Collaborative Innovation Center for Development of Veterinary Pharmaceuticals, College of Veterinary Medicine, Qingdao Agricultural University, Qingdao 266109, China; 15753637200@163.com (Z.H.); wxb200002@163.com (X.W.); yrm.cc@163.com (R.Y.); 3 Qingdao Vland Biotech Inc., Qingdao 266102, China; congyf@vlandgroup.com; 4 State Key Laboratory for Animal Disease Control, Harbin Veterinary Research Institute, Chinese Academy of Agricultural Sciences, Harbin 150069, China; wangjinliang@caas.cn

**Keywords:** novel goose parvovirus (NGPV), short beak and dwarfism syndrome (SBDS), pathogenicity, beak development index (BDI), cherry valley ducks

## Abstract

Duck short beak and dwarfism syndrome is a viral infectious disease caused by a novel goose parvovirus (NGPV), which results in growth retardation, short beaks, protruding tongues, and lameness in ducks. To determine whether short beak results from growth retardation or viral direct impact, this study sequenced the SBDSV-SD strain and investigated its pathogenicity in 2- and 8-day-old ducks. The formula model of beak development index was established by using the three-dimensional data of beak length, beak width and body weight of healthy ducks. The results show that phylogenetic analysis classified SBDSV-SD within the NGPV clade. The result of the challenge test shows that viral infection can cause weight loss, short beak, and viral shedding, and more severe symptoms in younger ducks. A beak development index (BDI), correlating beak length^2^ × width with body weight (R^2^ > 98%), was established. The application of BDI revealed a significant difference at two weeks post-infection in infected ducks, indicating that early short beak is attributable to a secondary injury of growth retardation rather than the direct impact of the virus. The BDI can effectively differentiate between these mechanisms.

## Simple Summary

Duck short beak and dwarfism syndrome (SBDS), caused by novel goose parvovirus (NGPV), leads to growth retardation, short beak, and lameness. In existing infection model studies, the common approach involves directly measuring the body weight as well as the length and width of the beak. However, this methodology fails to discriminate whether the beak development disorder results from a secondary phenomenon of delayed development or the direct impact of the virus. In this study, the pathogenicity of the newly isolated SBDSV—SD strain in Cherry Valley ducks was appraised. Subsequently, a three-dimensional formula model for the beak development index was constructed using the beak length, beak width, and body weight of healthy ducks. When this model was applied to infected ducks, it was found that this formula could effectively mirror the beak development disorder induced by the virus.

## 1. Introduction

The “Short Beak and Dwarfism Syndrome” (SBDS), also termed “duck macroglossia,” is a contagious disease caused by novel goose parvovirus (NGPV). SBDS is characterized by a mortality rate in ducks typically below 5%, whereas NGPV infection induces growth retardation, short beak, tongue protrusion, lameness, and increased bone fragility. Although the mortality rate of this disease is relatively low, the vast majority of infected ducks develop into “ numb ducks”, showing symptoms such as abnormal growth, stunted development, and weight loss [1]. The culling rate of such “ numb ducks” is as high as 80%, causing significant economic losses [2]. In addition, vertical transmission further complicates disease control [3].

NGPV is classified within the genus Dependovirus of the subfamily Parvovirinae. It is a single-stranded DNA virus with a diameter of 20–24 nm, exhibiting icosahedral symmetry. The viral genome is approximately 5.1 kb in length and contains inverted terminal repeats (ITRs) at both ends, as well as two open reading frames (ORFs). The left ORF encodes the non-structural proteins NS1 and NS2, while the right ORF encodes the structural proteins VP1, VP2, and VP3 [4,5].

SBDS was first reported in mule ducks in southern France (1971–1972), with subsequent outbreaks documented in Taiwan (1989), Poland (1995), and China (2015) [6,7,8]. Recent cases have emerged in Egypt [9] and Vietnam [10]. Previous studies on the pathogenicity of NGPV have shown that ducklings are highly susceptible to NGPV infection, which can cause dysplasia, short beak, tongue protrusion, and slope foot symptoms, and even lead to death [7,11,12]. However, there are obvious limitations in the assessment of beak development. Most studies have only been evaluated by direct comparison of absolute beak length or width, an approach that cannot distinguish whether developmental impairment of the beak is the result of a direct effect of the virus or merely a secondary concomitant phenomenon with systemic growth retardation (weight loss). The lack of a quantitative indicator to correlate beak development with general growth status is a key bottleneck for standardized assessment of pathological changes in this disease.

Inspired by comprehensive measures such as Body Mass Index (BMI = Weight/Height^2^) and the Ponderal Index (PI = Weight/Height^3^) [13,14,15,16,17,18,19] in human medicine, these indices correlate height and weight to assess nutritional and developmental status. We speculate that establishing a comprehensive index correlating beak size with body weight for ducks may provide a more scientific assessment of the specific effects of viruses on beak development.

In this study, we analyzed the genomic characteristics of an NGPV (SBDSV-SD) strain isolated in 2023 and investigated the pathogenicity of SBDSV-SD in 2- and 8-day-old Cherry Valley ducks. To this aim, we proposed and defined for the first time the beak development index (BDl), in order to provide new key indicators for establishing a standardized infection model.

## 2. Materials and Methods

### 2.1. Virus and Experimental Animals

The new goose parvovirus SBDSV-SD strain was originally isolated from the spleen of the cherry duckling flock with SBDS in Shandong province, China. The isolated virus was grown and titrated in 9-day-old embryonated Specific Pathogen Free (SPF) duck eggs through the allantoic cavities route. The titer of SBDSV-SD strain was calculated at 10^4.85^ ELD_50_/0.2 mL for intramuscular challenge in this study. One-day-old ducklings negative for maternal antibodies of NGPV were purchased from a hatchery in Qingdao. On the second day after hatching, we randomly assigned them to different groups based on their weight and housed them in separate, isolated rooms in a closed animal facility. They were fed high-quality commercial duck rations, and have unlimited access to water. The ducklings were identified by individually numbered leg rings.

### 2.2. Sequencing and Analysis of the NGPV Genome

The complete genome of SBDSV-SD strain was amplified by six conserved-region primer pairs (Table 1) and products were cloned into *pCE2* (Vazyme, Nanjing, China), and sequenced by Sangon Biotech (Shanghai, China). The genome sequences (PP236942.1) were compared with GenBank reference strains and generated neighbor-joining trees by MEGA 7.0 [20] (bootstraps 1000, p-distance, d: Transitions + Transversions).

### 2.3. Ethical Approval

All animal studies were conducted in accordance with the applicable animal welfare standards (Chinese National Standard (GB/T 42011-2022 [21])) and Declaration of Helsinki, and approved by the Institutional Ethics Committee of The National Animal Health Products For Engineering Technology Research Center (GCZX RD YF046-1.0, 25 November 2024).

### 2.4. Animal Experimental Design

Forty ducklings were randomly allocated into four groups (*n* = 10). One experimental group was infected with of NGPV at 2 days old, while the other group was infected at 8 days old. The infection dose is 0.5 mL per 100 g of body weight, and the ducklings of control groups were inoculated with equal PBS at the same age. Clinical symptoms such as listlessness, apathy, ruffled feathers, diarrhea, tongue protrusion, and lameness were monitored over a period of 21 days post-infection (dpi) or an extended period of 28 days post-infection (applicable only to Group 1 and Group 2). Pharyngeal and cloacal swabs were collected on days 0, 2, 4, 6, 10, 14, 21, and 28 (applicable only to Group 1 and Group 2) post-infection to assess viral shedding dynamics using Quantitative Real-Time PCR (qPCR). Body weight, beak length, and beak width were measured weekly (Table 2). At 30 days of age, the animals were humanely euthanized in accordance with ethical guidelines. During necropsy, tissue samples from the heart, liver, spleen, lung, kidney, pancreas, small intestine, beak, and tibia were collected and fixed in 4% formaldehyde solution for subsequent histopathological analysis. For molecular detection, 0.5 g of tissues from the heart, liver, spleen, lung, kidney, pancreas, small intestine, and thymus were homogenized in 0.5 mL of phosphate-buffered saline (PBS), followed by nucleic acid extraction. qPCR was performed to determine the toxin load in each tissue sample. Additionally, head, wing, and leg tissues were collected and subjected to radiological imaging examination.

### 2.5. NGPV Quantitative Real-Time PCR

Swabs/tissues were homogenized in PBS; DNA was extracted using Vazyme (Nanjing, China) Viral Kit 2.0 per manufacturer’s instructions. NGPV loads in duck samples were quantified using an NGPV qPCR assay. In brief, a 108 bp VP gene target was amplified using NGPV-F: AGTTCCTTTCCACAGCAT; NGPV-R: TTTCTGTTGCTGTCTACCTC; Probe: FAM-TTCGCTCATTCACAGGACTTAGACAGGC-BHQ1.Standard curves used *pUC19-NGPV VP2* dilutions (10^−1^–10^−10^; corresponding to 8.74 × 10^9^–8.74 × 10^−2^ copies/uL). Reactions (20 μL) contained 2 μL template, 0.4 μM primers, 0.2 μM probe, and All-Ready Mix(Qingdao Biotephy Co., Ltd., Qingdao, China). Cycling: 95 °C/3 min; 40 × [95 °C/10 s → 60 °C/30 s]. Specificity confirmed against 7 waterfowl pathogens; sensitivity: 8.74 copies/μL. Triplicate runs per sample.

### 2.6. Statistical Analysis

GraphPad Prism software version 8.02 (GraphPad Software Inc., San Diego, CA, USA) was utilized to perform correlation analyses and comparisons of collected data on body weight, beak length, beak width, swab viral shedding, and viral load across various tissues in both infected and control duck groups. Data were expressed as mean ± standard deviation (SD). Statistical significance was determined by Student’s *t* test analysis. *p* value < 0.05 was considered statistically significant between 2 groups. Normality of distribution was assessed by the Kolmogorov–Smirnov test.

### 2.7. Establishment of Beak Development Index (BDI)

Body weight, beak length, and beak width were measured weekly in a group of 20 healthy Cherry Valley ducks serving as blank controls over a three-week or four-week period, resulting in data collection at five consecutive time points. Refer to the different calculation formulas developed in the research on human body mass index [13], we examined the linear relationships between various morphometric indices of beak development—including beak length, beak width, the product of length and width (length × width), and the volumetric proxy (length^2^ × width)—and body weight. The relationship exhibiting the strongest correlation with body weight and satisfying the assumption of normality—assessed using the least squares method under a Gaussian model—was identified as the optimal predictor variable. Based on the relationship, a beak development index was formulated to quantitatively characterize beak growth relative to overall body size.

### 2.8. Application of Beak Development Index in Infected Ducks

The beak development index (BDI) values for both the infection group and the control group were calculated according to the established formula (BDI = beak length^2^ × width/body weight). GraphPad Prism software (version 8.02) was employed to compare and analyze the BDI between the two groups, in order to evaluate the potential impact of viral infection on beak development in ducks. Statistical significance was determined by Student’s *t* test analysis. *p* value < 0.05 was considered statistically significant.

## 3. Results

### 3.1. Genomic Characterization of SBDSV-SD

**Figure 1:**
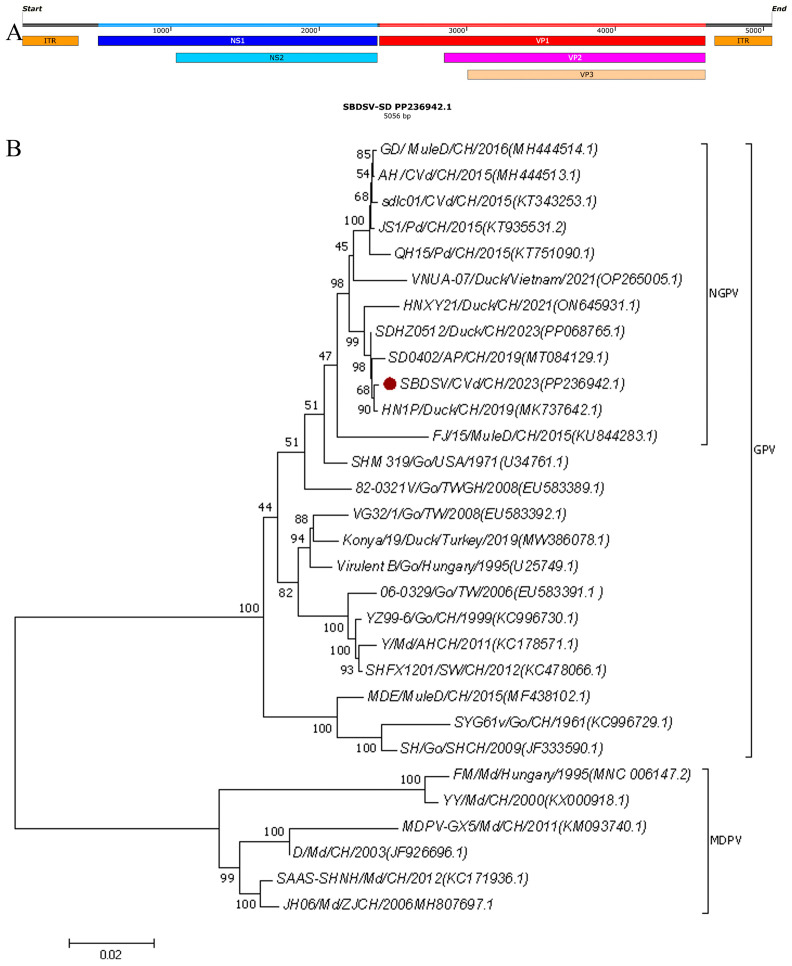
Genome structure and phylogenetic tree of NGPV strains obtained based on the genome nucleotide sequences. (**A**) Genome structure of NGPV; (**B**) phylogenetic tree of NGPV strains. Numbers at nodes indicate bootstrap percentages obtained after 1.000 replicates. Genotypes are indicated on the right side of the tree. Note: The red dot represents SBDSV-SD strain.

The length of SBDSV-SD genome is 5056 nucleotides, and 380 nucleotides inverted terminal repeats (ITRs) in the non-coding regions at both ends. Two major ORFs were identified: the ORF at 512~2395 nucleotides encodes the non-structural Rep protein (627 aa), while the ORF at 2414~4612 nucleotides encode VP1 (732 aa). VP2 and VP3 initiation codons are located, respectively, at 2849 and 3008 nucleotide (Figure 1A). The phylogenetic result shows that SBDSV-SD clusters with NGPV strains, formed a distinct clade with HN1P (2019), SD0402 (2019), and SDHZ0512 (2023) (Table 3), and diverged from the isolates before 2016 (Figure 1B).

### 3.2. Clinical Parameter Monitoring and Growth Metric Analysis

**Figure 2:**
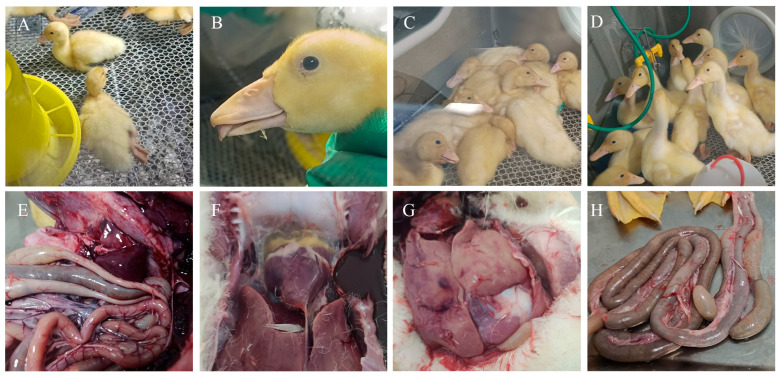
Clinical signs and gross lesions in NGPV infected ducks. (**A**) Leg fracture or lameness; (**B**) tongue extension; (**C**) listless, clustered and recumbency; (**D**) blank control. (**E**) splenic congestion; (**F**) pericardial effusion; (**G**) hepatomegaly; (**H**) intestinal flatulence.

**Figure 3:**
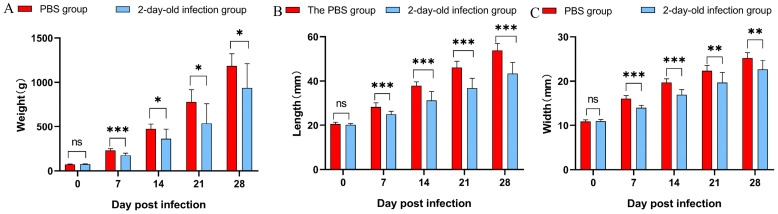
Body weight, beak length and beak width between PBS group and 2-day-old infection group. (**A**) Body weight; (**B**) length of beak; (**C**) width of beak. Note: ns represents *p* value > 0.05, no significant difference. “*” represents *p* value < 0.05; “**” represents *p* value < 0.01; “***” represents *p* value < 0.001.

In 2-day-old ducks, viral infection led to progressive clinical signs, with listlessness, anorexia, and recumbency appearing from day 4 post-infection, flaccid paralysis of the legs evident from day 7, and tongue protrusion observed starting on day 13. By day 28 post-infection, the recorded clinical manifestations included lameness (30%), tongue extension (20%), and developmental delay, which reached 100% incidence (Figure 2A–D). Gross pathological findings at necropsy revealed splenic hemorrhage (30%), pericardial effusion (20%), hepatomegaly (30%), and intestinal dilation (30%, Figure 2E–H). Compared with the control group, infected ducks exhibited a mean weight reduction of 24.24% ± 4.48% during the first four weeks post-infection (*p* < 0.05, Figure 3A). Beak length was significantly reduced by 17.22% ± 3.84% (*p* < 0.001, Figure 3B), and beak width decreased by 12.28% ± 1.17% (*p* < 0.05, Figure 3C).

**Figure 4:**
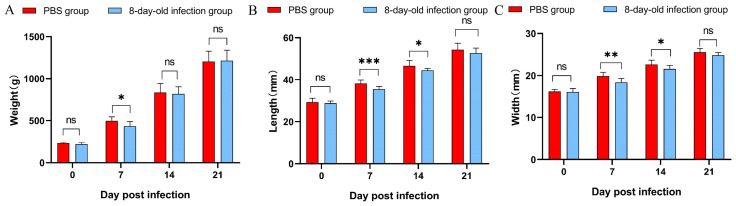
Body weight, beak length and beak width between PBS group and 8-day-old infection group. (**A**) Body weight; (**B**) length of beak; (**C**) width of beak. Note: ns represents *p* value > 0.05, no significant difference. “*” represents *p* value < 0.05; “**” represents *p* value < 0.01; “***” represents *p* value < 0.001.

The 8-day-old ducks exhibited temporary lethargy and recumbency from 1 to 3 dpi; no significant gross lesions were found during the post-mortem examination during the necropsy. The body weight was significantly reduced by 13.05% only at 1 week after infection (*p* < 0.05, Figure 4A). Additionally, beak length was significantly shorter by 7.05% and 4.74% at 1 and 2 weeks post-infection (*p* < 0.05, Figure 4B), and beak width was reduced by 7.97% to 4.63% at the same time (*p* < 0.05, Figure 4C). No significant differences were observed in these parameters at 3 weeks after the infection.

### 3.3. Histopathological Examination

**Figure 5:**
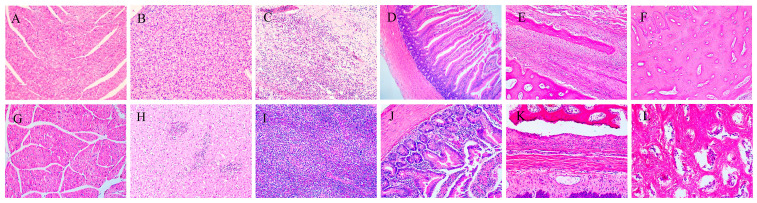
Microscopic lesions of ducks 28 days after NGPV infection (200×). (**A**–**F**) Infected ducks. (**A**) Heart: Granular degeneration of myocardial cells. (**B**) Liver: Hepatocyte swelling with numerous vacuoles in the cytoplasm and hepatocyte necrosis. (**C**) Spleen: No distinct splenic corpuscles, congestion, abundant fibrous-like substances, and infiltration of heterophilic granulocytes. (**D**) Duodenum: Partial necrosis of mucosal epithelial cells. (**E**) Beak: Abundant connective tissue in the bone tissue space, with visible trabeculae and bone marrow cavity in the middle of the bone tissue. There are many chondrocytes on the trabeculae and few osteoblasts on the edge of the bone marrow cavity. (**F**) Tibia: Chondrocytes are distributed in the bone tissue of the tibia, and no obvious osteoblasts are seen covering the inner surface of the bone marrow cavity. (**G**–**L**) Healthy ducks. (**G**) Heart. (**H**) Liver. (**I**) Spleen. (**J**) Duodenum. (**K**) Beak. (**L**) Tibia.

Histopathology examination revealed splenic fibrosis, hepatocellular necrosis, myocardial degeneration, intestinal mucosal necrosis, and chondrocyte proliferation in the beak and tibia, along with reduced osteoblast activity in the infected group (Figure 5).

### 3.4. Radiological Investigation

**Figure 6:**
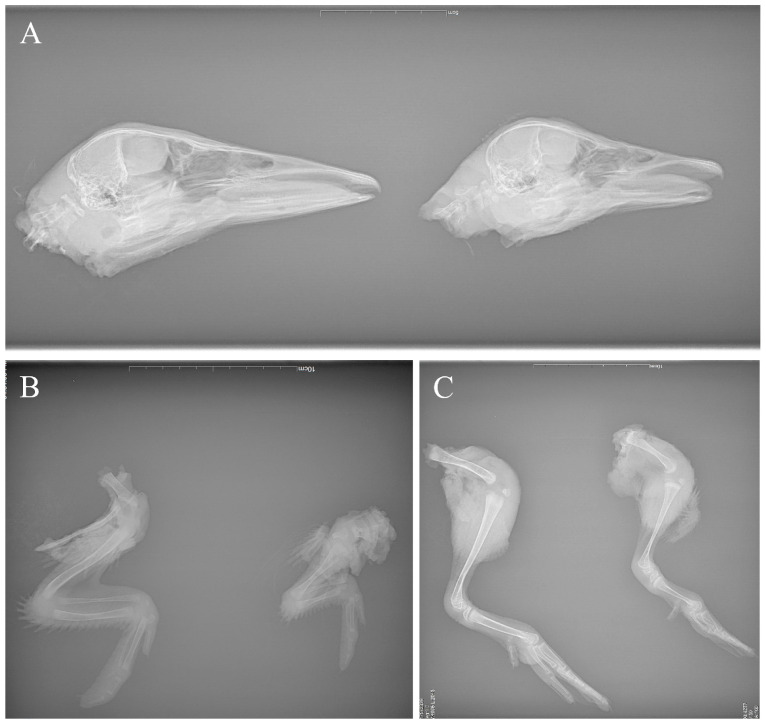
X-ray examination of ducks 28 days after NGPV infection. (**A**) Heads; (**B**) wings; (**C**) legs. Left, uninfected control group; right, infected group.

X-ray examination revealed severe hypoplasia of the bones in the beak, wings, and legs of the infected ducks, characterized by shortened bones, reduced mineral density, and diminished marrow cavity volume (Figure 6).

### 3.5. Viral Shedding and Tissue Tropism

**Figure 7:**
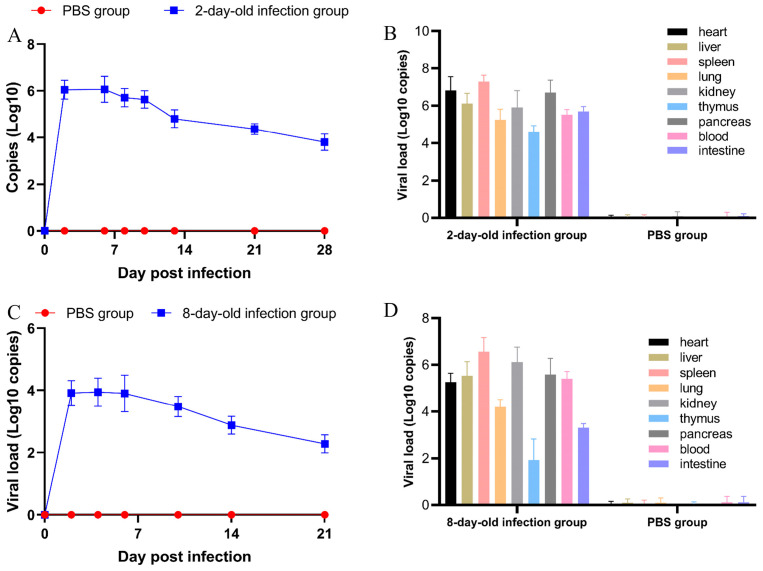
Viral qPCR results. (**A**) Virus shedding was monitored by qPCR in 2-day-old infection group; (**B**) the viral load of each organ in 2-day-old infection group; (**C**) virus shedding was monitored by qPCR in 8-day-old infection group; (**D**) the viral load of each organ in 8-day-old challenge group.

Viral DNA in oral and cloacal swabs were detected by NGPV qPCR after infection. In 2-day-old ducks, viral loads peaked at 10^6.08 ± 0.4^ copies/μL on 2 dpi, stabilizing until 6 dpi and declining to 10^3.80 ± 0.35^ copies/μL at 28 dpi (Figure 7A). Viral shedding in 8-day-old ducks exhibited similar curve patterns but demonstrated 100-fold lower titers, with a peak of 10^3.91 ± 0.4^ copies/μL (Figure 7C). Both 2-day-old and 8-day-old ducklings demonstrated significantly elevated viral loads in all examined organs compared to controls. Among the organs analyzed, the spleen exhibited the highest viral load, whereas the thymus displayed the lowest detectable levels (Figure 7B,D).

### 3.6. Establishment of Beak Development Index

**Figure 8:**
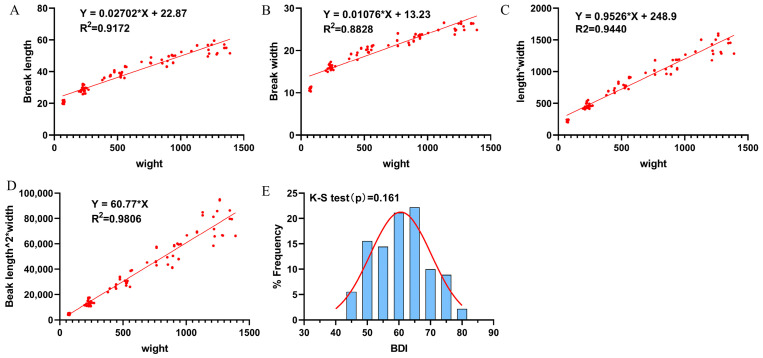
The linear analysis results of the degree of beak development and body weight. (**A**) Beak length; (**B**) beak width; (**C**) beak length× width; (**D**) beak length^2^ × width; (**E**) the normal distribution of the healthy duck BDI was confirmed by the Kolmogorov–Smirnov test. Note: The red line represents the fitting curve of the normal distribution, and the blue rectangles denote the frequency of data occurrence.

A total of 90 samples were collected at five time points. All samples exhibited a strong linear relationship (R^2^ > 0.8828, Figure 8A–D), with beak length^2^ × width showing the highest goodness of fit (R^2^ = 0.9806, Figure 8D). Based on these findings, we developed an index to evaluate beak development, termed the beak development index (BDI), calculated as BDI = (beak length^2^ × width)/body weight. Statistical analysis revealed that BDI values in healthy ducks followed a normal distribution, as confirmed by the Kolmogorov–Smirnov test (*p* value = 0.161, Figure 8E).

### 3.7. The Results of Beak Development Index Application in Infected Ducks

**Figure 9:**
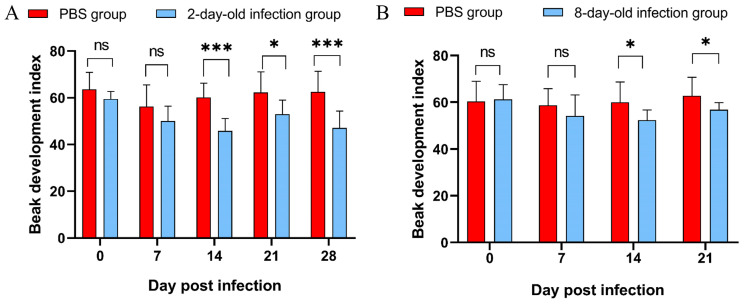
Beak development index between PBS group 2-day-old infection group and 8-day-old infection group. (**A**) Comparison between PBS group and 2-day-old infection group; (**B**) comparison between PBS group and 8-day-old infection group. Note: ns represents *p* value > 0.05, no significant difference. “*” represents *p* value < 0.05; “***” represents *p* value < 0.001.

Statistical analysis revealed that the beak development index in the 2-day-old and 8-day-old infection groups was significantly different from that of the control group two weeks after infection. Compared with the control group, 2-day-old infected ducks exhibited a mean BDI reduction of 21.32% ± 5.41% (*p* < 0.05, Figure 9A), while the 8-day-old infection group showed a reduction of 11.14% ± 2.05% (*p* < 0.05, Figure 9B).

## 4. Discussion

Since 2015, a novel duck-derived goose parvovirus (NGPV) has emerged on the mainland of China [8]. Despite its relatively low mortality rate, NGPV causes a high disability rate in ducks, resulting in significant economic losses for the waterfowl industry. Moreover, the disease can be transmitted vertically, posing a substantial challenge to disease prevention and control [3,12].

Recently, the NGPV virus has been detected in several countries including Poland [7,22], Egypt [9], and Vietnam [10], as well as China [8], where the incidence rate is particularly high. Genomic analyses of Chinese NGPV strains [8,23,24,25,26] reveal 96–100% sequence homology. In this study, the SBDSV-SD strain was clustered into the new goose parvovirus branch and shared 97.8–99.6% identity with 11 representative NGPV strains. Isolates HN1P and SD0402 in 2019 and SDHZ0512 in 2023 clustered into a new branch compared with the representative strains isolated in 2015.

The current pathogenicity studies on NGPV have confirmed that NGPV can infect mule ducks, Cherry Valley ducks, Peking ducks, etc. [7,11,12]. The common symptoms of NGPV are developmental delay, short beak, leg fracture and tongue extension, etc. For this study, we preferred to evaluate the pathological effects on Cherry Valley ducks, both because they are the most commonly used in meat duck breeding in China, and because the symptoms, previously listed and described in the literature, are more easily detectable. And our research results replicated the aforementioned clinical symptoms described. In addition, X-ray examination results of infected ducks revealed that the lengths of the mandible, humerus, ulna, metacarpal, femur, and metatarsal bones were significantly shorter compared to those in the control group. Microscopic pathological examination of the duck beaks and tibiae further demonstrated an increased number of chondrocytes and a reduced number of osteoblasts. These findings suggest that viral infection may disrupt the normal process of chondrocyte-to-osteoblast transformation, thereby impairing bone growth.

Furthermore, aforementioned methods for confirming the shortness of the beak are mostly through direct measurement of the beak length and width, which is unable to distinguish whether the abnormal development of the beak is caused by a viral infection or is a consequence of delayed development. Palya et al. [7] infected ducks of different ages with the Hungarian D176/02 strain and the ratio of beak length to beak width was introduced as a statistical indicator in the beak development analysis. Chen et al. [12] infected 1-day-old Cherry Valley ducks with the SDLC01 strain and conducted pathogenicity research. He merely briefly described the length of the beak, without conducting a significant difference analysis. And In Zhang’s and Xiao’s evaluation test of subunit vaccine immune protection, only the differences in body weight and beak length were measured [27,28]. Although there have been many studies on the pathogenicity or vaccine evaluation of NGPV in ducks, the evaluation of beak development has been limited to comparing the length and width of the beak, which raises the question of whether impaired beak development is directly caused by viral infection or secondary to reduced body weight growth. In this study, we explored a more scientific approach to characterizing the symptoms of beak developmental disorders caused by the virus using data-driven methods. Statistical analyses revealed a significant linear correlation between beak dimensions (length and width) and body weight, leading to the introduction of the “beak development index” as a novel metric for assessing beak abnormalities.

The BDI integrates multiple variables (beak length, beak width, and body weight) into a standardized index, which can more specifically reflect the development status of the beak and reduce the assessment deviation caused by simple body weight differences. The inspiration for this measurement standard comes from the human health index such as BMI and PI. Currently, this health index not only evaluates whether a person is overweight, but numerous studies have also linked it to the probability of contracting various diseases [17,29,30,31,32].

In this study, although there was a significant difference in beak length or width on the 7 dpi, the BDI showed a highly significant difference after 14 dpi. This indicates that the viral infection might initially affect overall growth before showing a specific inhibitory effect on beak development. Notably, the 8-day-old infection group demonstrated no significant differences in body weight or beak length and width at 21 dpi (Figure 4); although clinical recovery appeared to occur, qPCR results indicated ongoing viral replication and virus shedding in the swabs. The persistent presence of the virus may continue to impair beak development, a finding supported by the significant differences in the beak development index (BDI). Furthermore, it has been demonstrated that NGPV inhibits bone development in infected ducks through the BMP/Smad and FGF signaling pathways [33]. These findings suggest that the BDI may serve as a more reliable indicator for assessing beak development and enable more precise delineation of the temporal progression of NGPV infection. The variation pattern of the BDI aligns with this pathological process, indicating its potential as an effective tool for quantifying disease progression in future studies.

In this study, we used 20 healthy Cherry Valley ducks. We measured their body weight, beak length and beak width every week, and collected a total of 90 samples at five time points to establish a data model for beak development index. Although the data was statistically significant, the universality of BDI still needs to be further verified in larger-scale studies involving different species of ducks. Here, we introduce the concept and formula of the beak development index, which may serve as a foundation for researchers interested in this field to expand upon and validate using additional datasets. In the future evaluation of vaccine immunogenicity, we believe BDI will be used as a more sensitive indicator than singly beak length or width to investigate specific developmental disorders caused by NGPV.

## 5. Conclusions

In conclusion, we conducted a comprehensive pathogenicity study of NGPV in Cherry Valley ducks and, based on these findings, established the beak development index (BDI) for the first time, along with its preliminary application. BDI provides a novel method that can objectively reflect the impact of NGPV on the single element of beak development.

## Figures and Tables

**Table 1:** Primers used to amplify the complete nucleotide sequence of NGPV isolate SBDSV-SD.

Primers	Nucleotide Sequence (5′ to 3′)	Position	Length of Products (bp)
P1-F	CTTATTGGAGGGTTCGTTCG	1–20	191
P1-R	GCATGCGCGTGGTCAACCTAACA	169–191	
P2-F	GCATGCCGCGCGGTCAGCCCAAT	186–209	1294
P2-R	TCACCCAGCCGCACAGGATAC	1459–1480	
P3-F	TAGACAGGTGAAAGCTGCACT	1298–1319	1550
P3-R	TGATAGGCTCTTCTACTAGGC	2827–2848	
P4-F	GTAATCTTGGAAAGGCTGTAT	2773–2794	1515
P4-R	ATTTGCCATCAGTCTTCGGT	4268–4288	
P5-F	CCAGCATCCCAGCTCAGAATAGTTT	3994–4019	667
P5-R	ATATTTTGCGCGCCAGGAAGTGCTT	4636–4661	
P6-F	TCAATACTCTACAGGCCAGTGT	4418–4440	468
P6-R	CTGTTAGGTTGACCACGCGCATGC	4862–4886	

**Table 2:** The infection experimental designs used for the animal studies.

Group	Number	Day of Infection	Antigen	Dose	Tissue and Viscera Collection	Pharyngeal and Cloacal Swab Collection	Body Weight, Beak Length and Beak Width Collection
1	10	2-day-old	NGPV	0.5 mL per 100 g of body weight	4 weeks after infection	0 day before infection; 2, 4, 6, 10, 14, 21 and 28 days after infection	0 day before infection; 1, 2, 3 and 4 weeks after infection
2	10	2-day-old	PBS				
3	10	8-day-old	NGPV		3 weeks after infection	0 day before infection; 2, 4, 6, 10, 14 and 21 days after infection	0 day before infection; 1, 2, and 3 weeks after infection
4	10	8-day-old	PBS				

**Table 3:** Description of waterfowl parvovirus isolates involved in this study.

Viral Classification	Strain	Pathogenicity	Host	Geographic Origin	Collection Date	Genbank Accession No.
GPV	SYG61 v	Vaccine	Goose	China	1961	KC996729
GPV	SHM319	Pathogenic	Goose	USA	1971	U34761.1
GPV	82-0321	Pathogenic	Goose	Taiwan	1982	EU583390
GPV	YZ99-6	Pathogenic	Goose	China	1999	KC996730
GPV	06-0329	Pathogenic	Goose	Taiwan	2006	EU583391
GPV	VG32/1	Vaccine	Goose	Taiwan	2008	EU583392
GPV	82-0321v	Vaccine	Goose	Thailand	2008	U583389
GPV	SH	Pathogenic	Goose	China	2009	JF333590
GPV	Y	Pathogenic	Goose	China	2011	KC178571
GPV	SHFX1201	Pathogenic	SWAN	China	2012	KC478066
GPV	MDE	Pathogenic	mule duck	China	2015	MF438102
GPV	Konya/19	Pathogenic	Anser anser domesticus	Turkey	2019	MW386078
MDPV	FM	Pathogenic	Cairina moschata	Hungary	1995	U22967
MDPV	YY	Pathogenic	Muscovy duck	China	2000	KX000918
MDPV	D	Vaccine	Muscovy duck	China	2003	JF926696.1
MDPV	JH06	Pathogenic	Muscovy duck	China	2006	MH807697
MDPV	GX5	Pathogenic	Muscovy duck	China	2011	KM093740
MDPV	SAAS-SHNH	Pathogenic	Muscovy duck	China	2013	KC171936
NGPV	FJ15	Pathogenic	mule duck	China	2015	KU844283
NGPV	SDLC01	Pathogenic	Cherry Valley duck	China	2015	KT343253
NGPV	QH15	Pathogenic	Peking duck	China	2015	KT751090.1
NGPV	JS1	Pathogenic	Peking duck	China	2015	KT935531.2
NGPV	AH	Pathogenic	Cherry Valley duck	China	2015	MH444513.1
NGPV	GD	Pathogenic	mule duck	China	2016	MH444514
NGPV	SD0402	Pathogenic	Anas platyrhynchos	China	2019	MT084129
NGPV	HN1P	Pathogenic	Anas platyrhynchos	China	2019	MK737642
NGPV	HNXY21	Pathogenic	duck	China	2021	ON645931.1
NGPV	VNUA-07	Pathogenic	Duck	Vietnam	2021	OP265005.1
NGPV	SDHZ0512	Pathogenic	Duck	China	2023	PP068765.1
NGPV	SBDSV	Pathogenic	Cherry Valley duck	China	2023	PP236942.1

## Data Availability

The original contributions presented in this study are included in the article. Further inquiries can be directed to the corresponding authors.
